# Aldehyde dehydrogenase 2 activation ameliorates CCl_4_‐induced chronic liver fibrosis in mice by up‐regulating Nrf2/HO‐1 antioxidant pathway

**DOI:** 10.1111/jcmm.13677

**Published:** 2018-05-25

**Authors:** Xin Ma, Qin Luo, Hong Zhu, Xuejing Liu, Zhen Dong, Kaili Zhang, Yunzeng Zou, Jian Wu, Junbo Ge, Aijun Sun

**Affiliations:** ^1^ Institute of Biomedical Sciences Fudan University Shanghai China; ^2^ Department of Cardiology Shanghai Institute of Cardiovascular Diseases Zhongshan Hospital Fudan University Shanghai China; ^3^ Department of Medical Microbiology School of Basic Medical Sciences Fudan University Shanghai China; ^4^ Shanghai Institute of Liver Diseases Fudan University Shanghai Medical College Shanghai China; ^5^ Shanghai Cardiovascular Medical Center Fudan University Shanghai China; ^6^ Institute of Pan‐vascular Medicine Fudan University Shanghai China

**Keywords:** Alda‐1, ALDH2, CCl_4_, liver fibrosis, mitophagy, Nrf2/HO‐1

## Abstract

Mitochondrial aldehyde dehydrogenase 2 (ALDH2) is critical in the pathogenesis of alcoholic liver cirrhosis. However, the effect of ALHD2 on liver fibrosis remains to be further elucidated. This study aimed to demonstrate whether ALDH2 regulates carbon tetrachloride (CCl_4_)‐induced liver fibrosis and to investigate the efficacy of Alda‐1, a specific activator of ALDH2, on attenuating liver fibrosis. ALDH2 expression was increased after chronic CCl_4_ exposure. ALDH2 deficiency accentuated CCl_4_‐induced liver fibrosis in mice, accompanied by increased expression of collagen 1α1, α‐SMA and TIMP‐1. Moreover, ALDH2 knockout triggered more ROS generation, hepatocyte apoptosis and impaired mitophagy after CCl_4_ treatment. In cultured HSC‐T6 cells, ALDH2 knockdown by transfecting with lentivirus vector increased ROS generation and α‐SMA expression in an in vitro hepatocyte fibrosis model using TGF‐β1. ALDH2 overexpression by lentivirus or activation by Alda‐1 administration partly reversed the effect of TGF‐β1, whereas ALDH2 knockdown totally blocked the protective effect of Alda‐1. Furthermore, Alda‐1 administration protected against liver fibrosis in vivo, which might be mediated through up‐regulation of Nrf2/HO‐1 cascade and activation of Parkin‐related mitophagy. These findings indicate that ALDH2 deficiency aggravated CCl_4_‐induced hepatic fibrosis through ROS overproduction, increased apoptosis and mitochondrial damage, whereas ALDH2 activation through Alda‐1 administration alleviated hepatic fibrosis partly through activation of the Nrf2/HO‐1 antioxidant pathway and Parkin‐related mitophagy, which indicate ALDH2 as a promising anti‐fibrotic target and Alda‐1 as a potential therapeutic agent in treating CCl_4_‐induced liver fibrosis.

## 1 INTRODUCTION

Liver fibrosis is a consequence of wound‐healing process responded to several types of acute or chronic liver injury, including ethanol consumption, viral infection, fatty liver disease and metabolic disorders.^1^, ^2^, ^3^ Although early‐phase liver fibrosis is considered a reversible pathological process, late‐stage liver fibrosis will deteriorate to cirrhosis, portal hypertension or even hepatocellular carcinoma, which lead to increased morbidity and mortality.^4^, ^5^ Carbon tetrachloride (CCl_4_) is a widely used toxicant in experimental animals to induce liver lesion and liver fibrosis.^6^ Chronic CCl_4_ exposure causes collagen deposition, hepatocellular damage, oxidative stress and activation of hepatic stellate cells (HSCs).^7^ Activated HSCs promote syntheses of extracellular matrix proteins and expression of tissue inhibitor of metalloproteinase (TIMP), increase cell migration and proliferation, and enhance the fibrogenic capacity.^8^, ^9^

Oxidative stress and reactive oxygen species (ROS) generated during toxicant metabolism was proved to be involved in the activation of HSCs and the progression of liver fibrosis.^10^, ^11^ Several antioxidant systems are involved in the protection against oxidative stress under physiological and pathological conditions. Nuclear factor erythroid 1‐related factor 2 (Nrf2) is a nuclear transcription factor which binds to antioxidant response element (ARE) and participates in regulating transcription and expression of antioxidant enzymes.^12^ Previous study has proved that activation of Nrf2 could alleviate the progression of liver fibrosis.^13^, ^14^ Heme oxygenase‐1 (HO‐1) is an antioxidant enzyme which is commonly up‐regulated upon the stimulation of oxidative stress and injury.^15^ Notably, Nrf2 can directly regulate the expression of HO‐1,^16^, ^17^, ^18^ which shed light on the role of Nrf2/HO‐1 pathway in protecting against further hepatic damage.^16^ In addition, autophagy/mitophagy could serve as a cellular protective mechanism in attenuating ROS overproduction and mitochondrial damage in several liver diseases.^19^, ^20^ Therefore, inhibition of ROS generation could provide a potential therapeutic target for attenuating hepatic fibrosis.

Aldehyde dehydrogenase 2 (ALDH2) is a key enzyme that metabolizes reactive acetaldehyde into non‐toxic acetic acid, and detoxifies ROS‐generated aldehyde substances such as 4‐hydroxy‐2‐nonenal (4‐HNE) and malondialdehyde (MDA).^21^, ^22^ Nearly, half of the East‐Asian population possessed the genetic mutation which results in the deficiency of ALDH2 activity.^23^, ^24^ Findings from our laboratory and others revealed a beneficial role of ALDH2 in rescuing against certain diseases, such as ischemia heart disease, alcoholic liver disease and stroke,^25^, ^26^ which might be mediated through regulating oxidation stress, apoptosis and autophagy.^27^, ^28^, ^29^, ^30^, ^31^ Nevertheless, the role of ALDH2 in CCl_4_‐induced chronic liver fibrosis and the underlying mechanisms remains to be further elucidated. To this end, the present study was designed to investigate the effects of ALDH2 on CCl_4_‐induced liver fibrosis, collagen deposition and oxidative stress. Furthermore, Alda‐1, a specific ALDH2 activator, was administered to further demonstrate the therapeutic effect of ALDH2 activation on liver fibrosis.

## 2 MATERIALS AND METHODS

### 2.1 Animals and groups

Adult male C57BL/6 (8‐week old) was purchased from Shanghai Animal Administration Centre (Shanghai, China) and ALDH2 knockout mice with the same genetic background were reproduced as described previously.^32^ For the wild‐type (WT) and ALDH2 knockout (KO) group, age‐ and sex‐matched mice received twice‐weekly intraperitoneal injections with CCl_4_ (0.32 mL/kg; Sigma‐Aldrich, St. Louis, MO, USA) for 8 weeks. CCl_4_ solution was prepared as 1.0 mg/mL in corn oil (Sigma Aldrich). The control group was intraperitoneally injected with corn oil according to the bodyweight. For the ALDH2 activator group, age‐ and sex‐matched WT mice were treated with 5 mg/kg Alda‐1 (Tocris Bioscience, USA). Alda‐1 was intraperitoneally injected 24 hours ahead of CCl_4_ injection, which was dissolved in DMSO at a concentration of 1 mL/kg. The Alda‐1 control group was intraperitoneally injected with oil plus DMSO. During the experiment, the survival rate of mice was recorded. All animal experiments were performed in accordance with the National Institutes of Health Guide for the Care and Use of Laboratory Animals (NIH Pub. No. 86‐23, revised 1996) and with the approval of the Institutional Animal Care and Use Committee of Fudan University, China.

### 2.2 Samples and parameters

Mice were submitted an 8‐week CCl_4_ exposure and killed under deep anaesthesia with sodium pentobarbital (150 mg/kg, i.p.). The serum was collected by centrifugation for biochemical analysis. ALT and AST were determined with biochemical kits which were purchased from Nanjing Jiancheng Bioengineering Institute (Nanjing, China). Livers were photographed and weighted to calculate liver/body ratio.

### 2.3 Histopathological and immunohistochemical analyses

Livers were arrested and rapidly rinsed with PBS and then immersed into 4% paraformaldehyde or OCT compound (Tissue‐Tek; Sakura Finetek Europe B.V., Alphen aan den Rijn, the Netherlands). Specimens were fixed in 4% paraformaldehyde for 2‐3 days, embedded in paraffin, serially sectioned (4 μm) and stained with haematoxylin eosin (H&E), Masson's trichrome to assess liver morphology and liver collagen content, respectively. For the immunohistochemical study of collagen content, anti‐alpha‐smooth muscle actin (α‐SMA) antibody (Sigma‐Aldrich; 1:100 dilution) was used. Frozen specimens and HSC‐T6 cells were stained with dihydroethidium (DHE, Eugene, OR) for 30 minutes at 37°C to detect liver ROS production, respectively. Images were analysed using Image‐Pro Plus software (Media Cybernetics, Rockville, MD, USA).

### 2.4 Real‐time PCR assay

Samples of total RNA from mouse liver tissues were extracted by Trizol reagent (Life Technologies). RNA concentration was quantitated by measuring the absorbance at the 260 and 280 nm with a spectrophotometer (NanoDrop Technologies, Wilmington, DE, USA). About 500 ng of RNA was reverse transcribed into cDNA in a 20 μL reaction volume using PrimeScript™ RT Master Mix kit (TaKaRa Biotechnology Co., Ltd.) according to the manufacturer's manuals. The mRNA expressions were quantified by real‐time PCR with SYBR Premix Ex Taq II (Tli RNaseH Plus) using a Real‐Time PCR System (Bio‐Rad, Hercules, CA, USA). The mRNA level of endogenous GAPDH was used as an internal control. Primer sequences were looked up at PrimerBank database and synthetized by Sangon Biotech Co., Ltd. (Shanghai, People's Republic of China).

### 2.5 Western blot analyses

Samples containing equal amounts of proteins were separated by 10% or 12% sodium dodecyl sulphate polyacrylamide gel electrophoresis (SDS‐PAGE) and transferred to polyvinylidene fluoride (PVDF) membranes (Millipore) in an ice bath. The membranes were blocked with 5% bovine serum albumin (BSA) in TBST for l hour at room temperature. The membrane was washed with TBST and then cut into stripes accordingly. Stripes were then incubated overnight at 4°C with primary antibodies Anti‐Nrf2 (1:1000; Abcam), Anti‐HO‐1 (1:10 000; Abcam), Anti‐ALDH2 (1:5000; Novus Biology), Anti‐Bcl‐2 (1:1000; Cell Signaling Technology), Anti‐Bax (1:1000; Cell Signaling Technology), Anti‐LC3B (1:1000; Cell Signaling Technology), Anti‐p62 (1:1000; Cell Signaling Technology) and Anti‐α‐SMA (1:2000; Sigma‐Aldrich). Then, the stripes were incubated with secondary antibodies goat‐anti‐rabbit IgG (H+L) or goat‐anti‐mouse IgG (H+L) for 1 hour at room temperature. Finally, the blot was scanned by unfiltered rays with the Odyssey Imaging System (LI‐COR, Lincoln, NB, USA), and bands were quantified by Image J software (National Institutes of Health).

### 2.6 Cell culture

HSC‐T6 cell line was purchased from TongPai Biological Technology co., Ltd (Shanghai, China). The cells were cultured in Dulbecco's Modified Eagle's Medium (DMEM) supplemented with 10% FBS (GIBCO, Invitrogen, Carlsbad, CA, USA), 100 IU/mL penicillin and 100 mg/mL streptomycin. Cells were maintained at 37°C in a humidified air containing 5% CO_2_ for experiments. The passages of 2 and 4 were used in our experiment. Lenti viral vectors (Hanyin Co, Shanghai, China) were employed to deliver ALDH2 or to inhibit the expression of ALDH2 in the HSC‐T6 cell line. Cells were cultured in 6‐well plates (10^6^ cells per well) in 2 mL of culture medium and treated with TGF‐β1 (15 μg/mL; Peprotech, Inc., Rocky Hill, NJ, USA) and/or Alda‐1 (20 μmol/L) vs vehicle (0.1% DMSO).

### 2.7 Statistical analyses

The data are presented as the means ± standard error of mean (SEM) and compared with Student's independent *t* test to analyse 2 groups. One‐way ANOVA was performed to analyse multiple groups, followed by LSD analysis. The survival curve was made with GraphPad Prism 5 and the other data were analysed with SPSS software (version 18.0). Values of *P *<* *.05 were considered statistically significant.

## 3 RESULTS

### 3.1 ALDH2 deficiency deteriorates CCl_4_‐induced liver function

**Figure 1:**
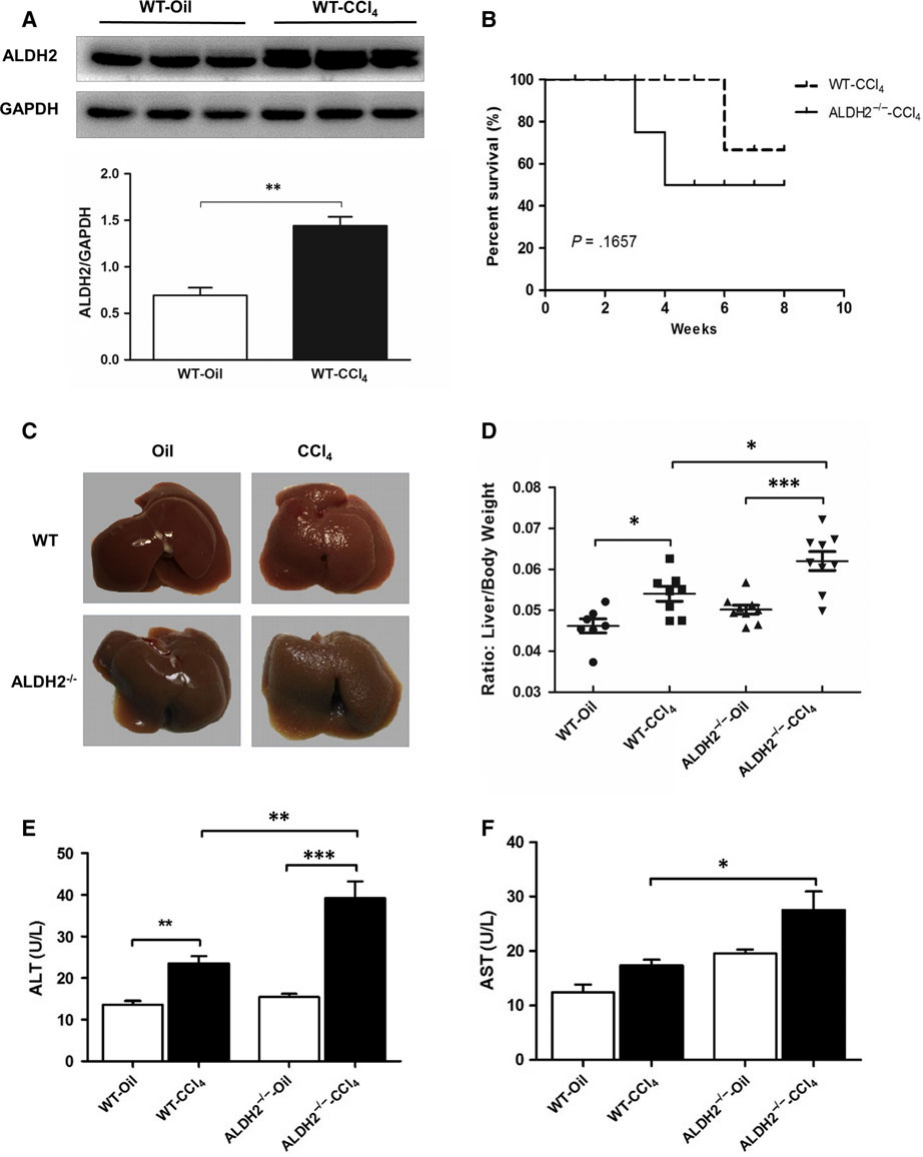
ALDH2^−/−^ mice manifested worse hepatic function after CCl_4_ treatment. A, Western blot results of the ALDH2 expression after CCl_4_ treatment; B, Survival rate; C, Representative photographs of liver; D, Liver/bodyweight ratio; E, F, Serum ALT and AST levels. The values represent means ± SEM (n = 7‐9). *P <* *.05, **P <* *.01, ***P <* *.001

The experimental model of liver fibrosis in mice was induced by intraperitoneally injection of CCl_4_ (0.32 mg/kg) for 8 weeks. Western blotting result showed an increased ALDH2 expression after CCl_4_ treatment (Figure 1A). Then, we used WT and ALDH2^−/−^ mice to further detect the role of ALDH2 in CCl_4_‐induced liver fibrosis. During the treatment, no death occurred in the controlled WT and ALDH2^−/−^ mice. However, 1 mouse died in WT‐CCl_4_ group, and 3 mice died in ALDH2^−/−^‐CCl_4_ group (Figure 1B). As shown in Figure 1C, CCl_4_ treatment induced a rough and granular liver surface in WT mice, which was worse in ALDH2^−/−^ mice. Additionally, CCl_4_ treatment resulted in an increased liver/bodyweight ratio in WT mice, which was further increased in ALDH2^−/−^ mice (Figure 1D). Liver injury was further detected by measuring plasma alanine aminotransferase (ALT) and aspartate aminotransferase (AST) levels. The results showed that ALT level was significantly increased in CCl_4_‐induced WT mice compared with the control mice, which was further increased in ALDH2^−/−^‐CCl_4_ group (Figure 1E). Like ALT, AST level was increased in WT and ALDH2^−/−^ mice after CCl_4_ treatment (no statistical significance), and ALDH2^−/−^‐CCl_4_ group showed a higher level than WT‐CCl_4_ group (Figure 1F). Taken together, the above data indicate that ALDH2 deficiency deteriorates hepatic function in the CCl_4_‐induced chronic liver injury mouse model.

### 3.2 ALDH2 deficiency accentuates hepatic fibrosis and collagen deposition

**Figure 2:**
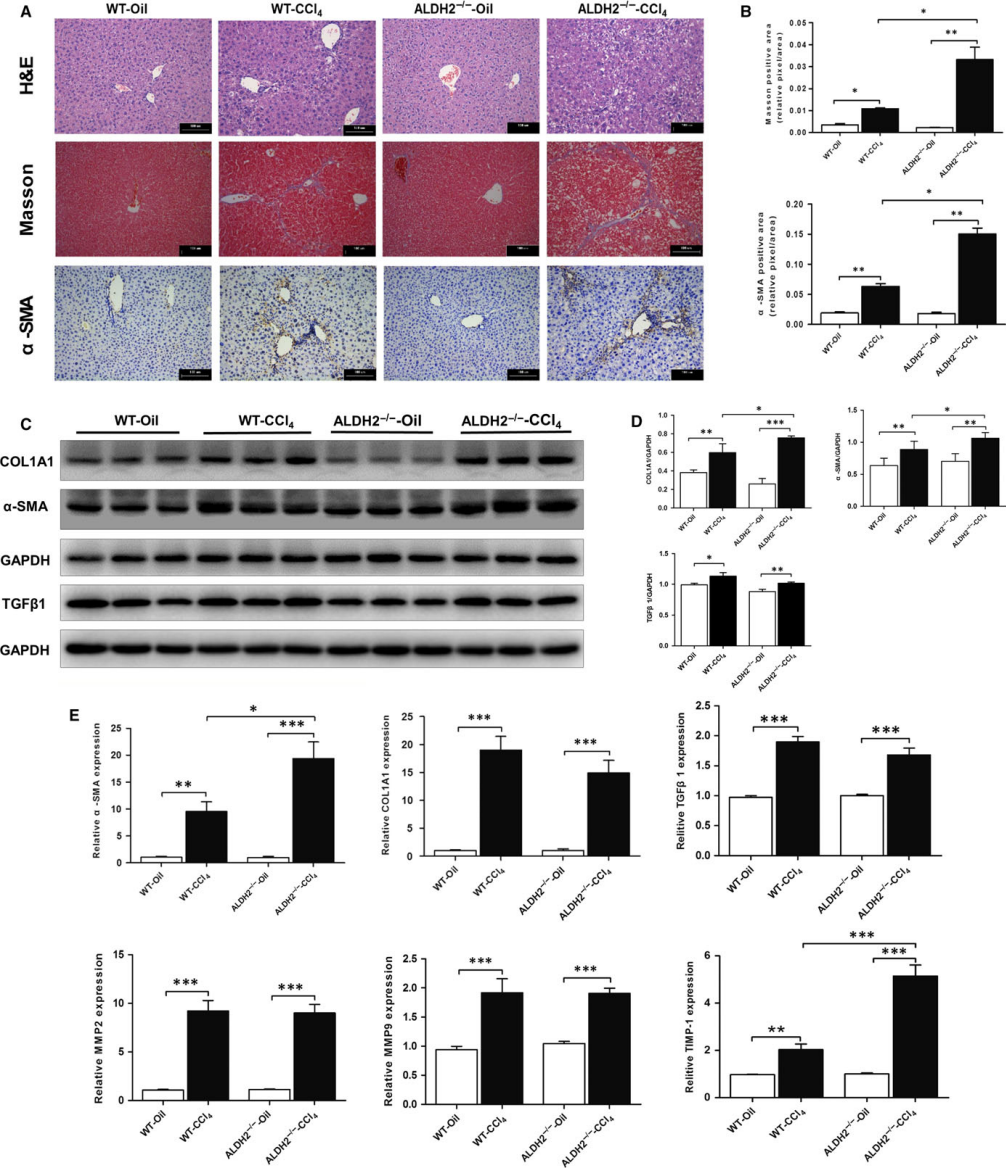
ALDH2 deficiency deteriorates hepatic fibrosis along with overproduction of extracellular matrix. A, Representative histopathological pictures of the liver. Upper panel: Representative H&E staining images. Middle panel: Representative Masson's trichrome staining images. Lower panel: Representative images of α‐SMA staining. B, Quantification of Masson's trichrome and α‐SMA‐positive area. C, Representative western blot pictures of proteins COL1A1, α‐SMA and TGFβ1. D, Western blot analyses of COL1A1, α‐SMA and TGFβ1. E, Real‐time PCR analyses of profibrotic genes. The values represent means ± SEM (n = 7‐9). *P <* *.05, **P <* *.01, ***P <* *.001. Scare bar, 100 μm

To examine the effect of ALDH2 deficiency on CCl_4_‐induced chronic liver fibrosis, H&E staining, Masson staining and immunohistochemistry staining were used. In the oil‐controlled WT and ALDH2^−/−^ groups, H&E staining showed normal lobular structure with central veins, radiating hepatic cords and little collagen fibres around some vessels, without inflammation and necrosis area. However, CCl_4_ administration induced focal hepatic necrosis and increased numbers of inflammatory cells accompanied by dilated blood sinusoids and congested central veins in WT mice, which was further deteriorated in ALDH2^−/−^ mice (Figure 2A). Liver fibrosis was then determined by performing Masson's trichrome and alpha smooth muscle actin (α‐SMA) staining. As shown in Figure 2A, in the paired‐control groups, WT and ALDH2^−/−^ mice had comparable levels of collagen (blue) and α‐SMA‐positive (brown) staining. However, in the CCl_4_‐induced groups, ALDH2^−/−^ mice showed much more abundant collagen deposition and α‐SMA‐positive staining than WT mice. To further examine the effect of ALDH2 deficiency on hepatic fibrogenesis, western blot and real‐time PCR analysis were used to examine α‐SMA, collagen 1α1 (I), TGF‐β1, MMP2, MMP9 and TIMP‐1 expressions. As illustrated in Figure 2C,D, CCl_4_ induced a significant increase in α‐SMA and collagen 1α1 (I) expression in the protein level, which was further increased in ALDH2^−/−^ mice. The protein expression of TGF‐β1 was also increased after CCl_4_ treatment both in WT and ALDH2^−/−^ mice, whereas no difference was found between them. Additionally, RT‐PCR analyses showed significantly increased hepatic expressions of α‐SMA, collagen 1α1 (I), TGF‐β1, MMP2, MMP9 and TIMP‐1 after CCl_4_ treatment both in WT and ALDH2^−/−^ mice (Figure 2E). Among them, the mRNA expressions of α‐SMA and TIMP‐1 were further increased in ALDH2^−/−^ mice. Taken together, our data demonstrated that ALDH2 deficiency may cause deteriorated hepatic fibrosis with markedly increased collagen deposition and profibrotic mRNA expression.

### 3.3 ALDH2 deficiency cause more ROS production in response to CCl_4_ administration

**Figure 3:**
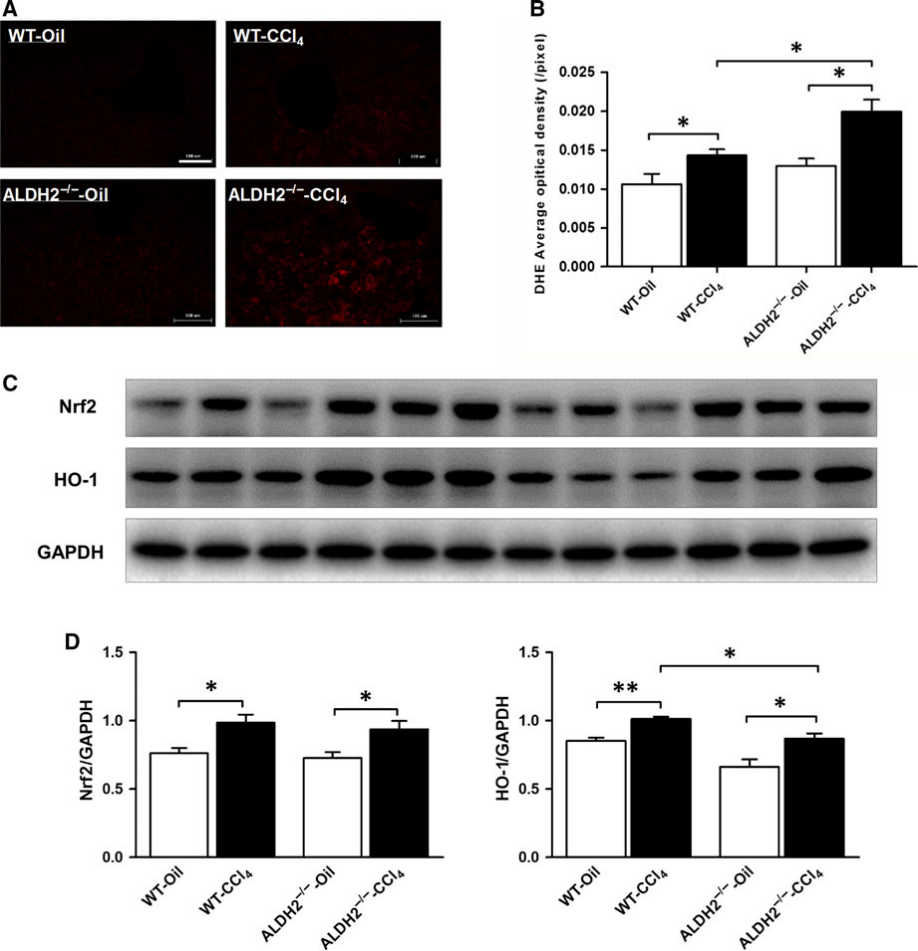
ALDH2^−/−^ mice showed more ROS generation in response to CCl_4_ administration. A, Representative images of DHE staining. B, Quantification of DHE density. The values represent means ± SEM. (n = 7‐9) *P <* *.05. Scare bar, 100 μm. C, Representative western blot pictures of Nrf2 and HO‐1. D, Western blot analyses of Nrf2 and HO‐1

Since hepatic oxidative stress is involved in the development of liver fibrosis, we measured CCl_4_‐induced ROS production using DHE staining among the 4 groups. As shown in Figure 3A,B, barely no positive DHE staining could be found in the liver of oil‐treated mice; however, much more positive DHE staining was found after CCl_4_ treatment both in WT and ALDH2^−/−^ mice. Furthermore, ALDH2^−/−^‐CCl_4_ mice showed more positive DHE staining in the liver compared with WT‐CCl_4_ mice. Since Nrf2/HO‐1 cascade was proved to be involved in attenuating oxidative stress in liver fibrosis, we detected the expression level Nrf2/HO‐1. Interestingly, both Nrf2 and HO‐1 increased significantly after CCl_4_ treatment, whereas only HO‐1 was found to be partly reversed in ALDH2 knockout mice. These results revealed that ALDH2 deficiency further increase CCl_4_‐induced ROS production in the liver, which might be partly through down‐regulating the antioxidant signalling.

### 3.4 ALDH2^−/−^ deficiency induces hepatic apoptosis while inhibits mitophagy

**Figure 4:**
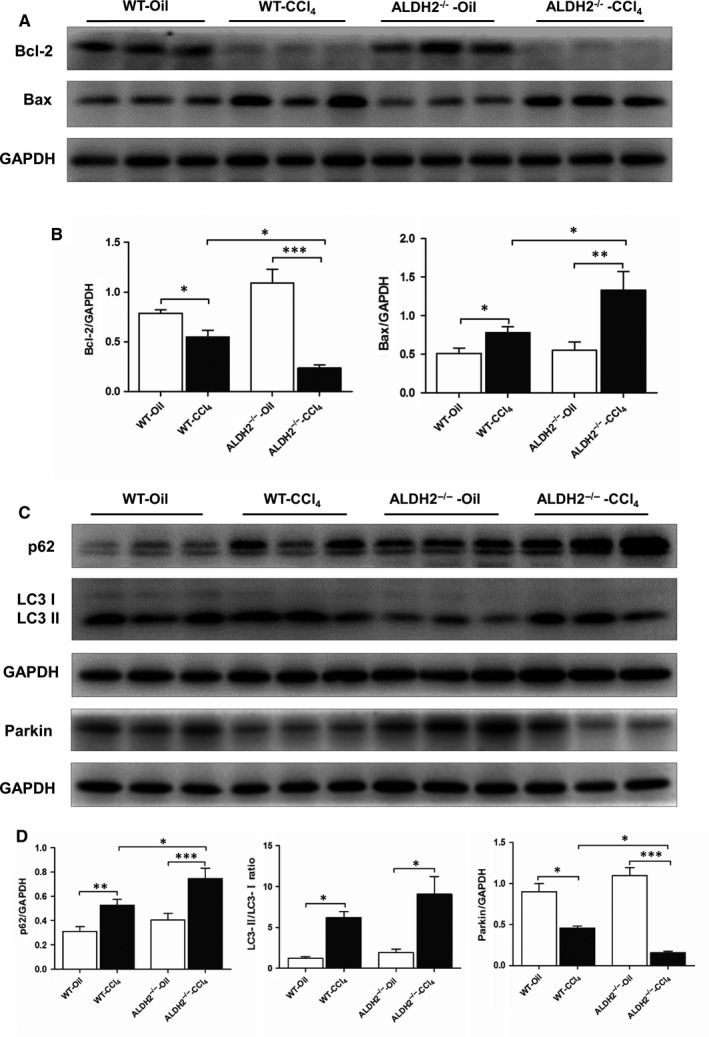
ALDH2^−/−^ deficiency accelerates the CCl_4_‐induced apoptosis while inhibits mitophagy. A, Representative western blot pictures of apoptosis‐related proteins Bcl‐2 and Bax. B, Quantification analyses of Bcl‐2 and Bax. C, Representative western blot pictures of mitophagy‐related proteins p62, LC3B and Parkin. D, Quantification analyses of the expression of p62, LC3B and Parkin. The values represent means ± SEM (n = 7‐9). *P <* *.05, **P <* *.01, ***P <* *.001

Hepatic apoptosis and mitophagy are both involved in the pathogenesis of liver fibrosis. To investigate the effect of ALDH2 deficiency on hepatic apoptosis, the expression levels of anti‐apoptotic protein Bcl‐2 and pro‐apoptotic protein Bax were detected in the liver tissue in vivo. The results revealed that Bax was markedly increased, whereas Bcl‐2 decreased after CCl_4_ treatment both in WT and ALDH2^−/−^ mice. Furthermore, compared with WT mice, ALDH2 deficiency further amplified these differences showed as significantly increased Bax and decreased Bcl‐2 levels upon CCl_4_ induction (Figure 4A,B). To further investigate the effect ALDH2 deficiency on hepatic mitophagy, protein expression levels of p62, LC3, and Parkin were examined. The expression level of p62 protein, which is a polyubiquitin‐binding protein sequestered and degraded during autophagy, significantly increased after CCl_4_ treatment compared to that of the control group. While ALDH2 deficiency further increased the expression of p62. In addition, CCl_4_ administration significantly increased the ratio of LC3‐II to LC3‐I, which was further increased in the ALDH2^−/−^ mice (no significance). These data indicated that CCl_4_ metabolism induced an autophagic flux impairment, and ALDH2 deficiency further deteriorated the impairment. As a mitochondrial membrane protein, Parkin is involved in the initiation of mitophagy. As shown in Figure 4, the protein expression of Parkin was significantly decreased after CCl_4_ treatment, which was further decreased in ALDH2^−/−^ mice. Taken together, these data suggested that CCl_4_ administration promotes hepatic apoptosis and deteriorates autophagy/mitophagy, leading to impaired hepatic function, while ALDH2 deficiency further accentuates the deteriorative effect of CCl_4_.

### 3.5 ALDH2 activation by Alda‐1 blocks TGF‐β1‐induced HSC‐T6 cell activation through attenuating ROS generation

**Figure 5:**
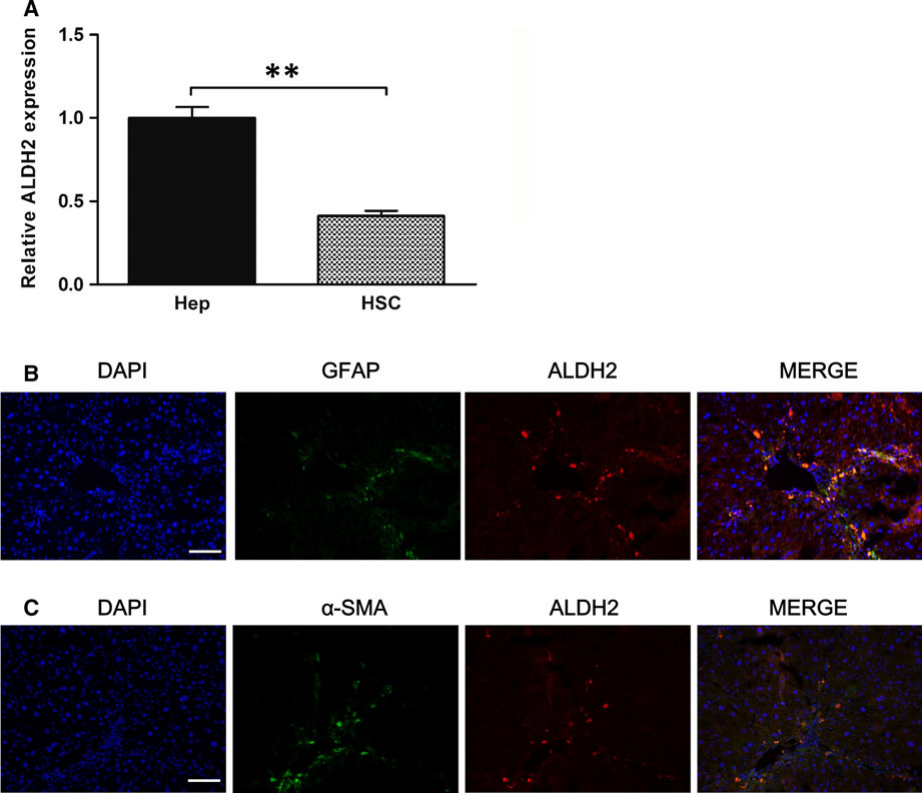
ALDH2 expression in hepatocytes and HSCs. A, Real‐time PCR analyses of relative ALDH2 expression. The values represent means ± SEM (n = 3). **P <* *.01. B, Representative immunofluorescence pictures of GFAP and ALDH2 co‐localization staining. C, Representative immunofluorescence pictures of α‐SMA and ALDH2 co‐localization staining

**Figure 6:**
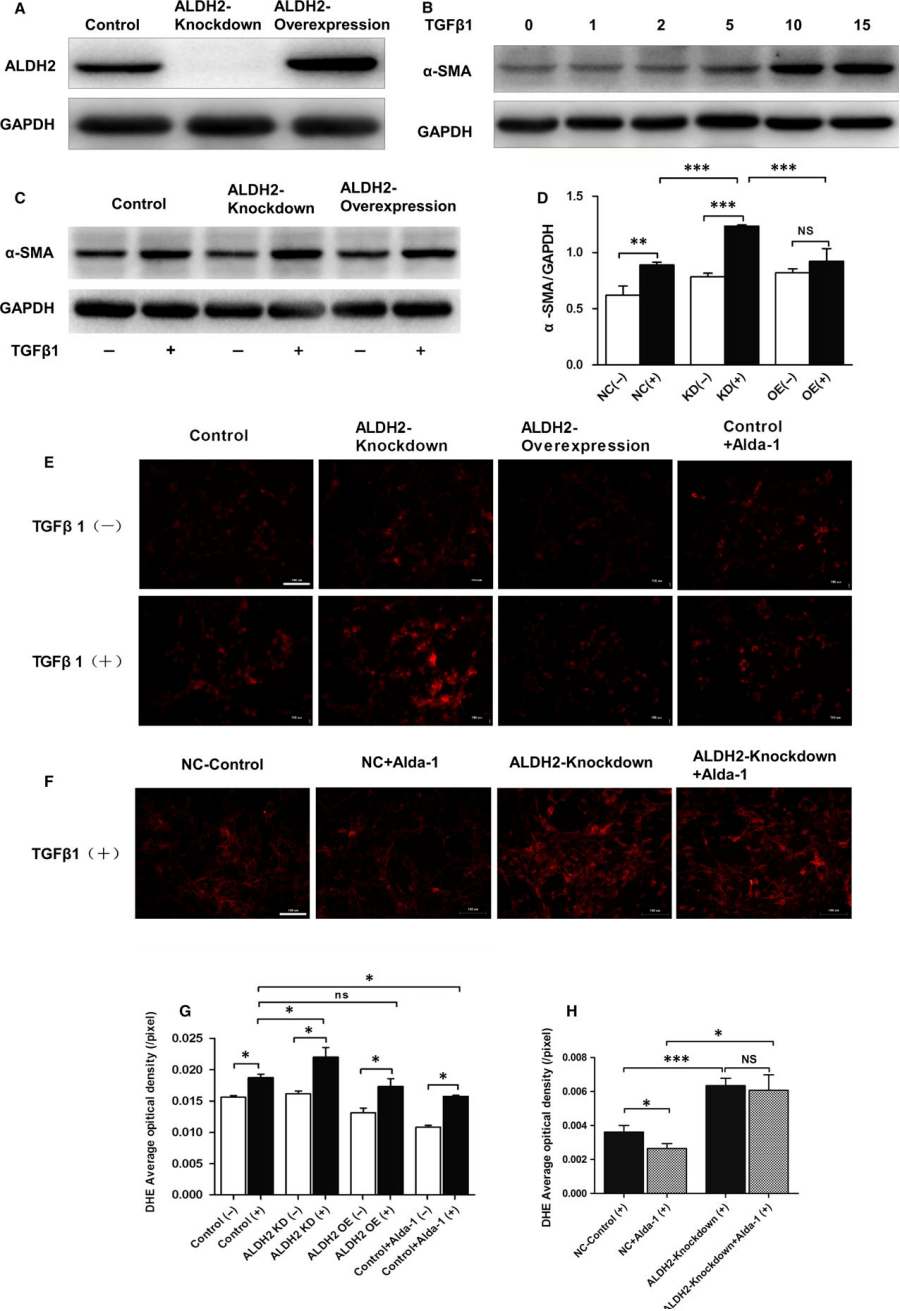
ALDH2 activation reduced TGF‐β1‐induced HSC‐T6 cell activation along with attenuation of ROS generation. A, Representative western blot pictures of rat hepatic stellate cells transfected with scramble, ALDH2 knockdown and ALDH2 overexpression lentiviral vectors. B, Western blot analyses of α‐SMA indicated HSC‐T6 activation induced by a variety concentration of TGF‐β1. C, Representative western blot pictures of α‐SMA with or without TGF‐β1 administration. D, Quantification analyses of the expression of α‐SMA. E, Representative DHE staining with or without TGF‐β1 administration. F, Representative DHE staining with or without Alda‐1 administration. G, H, Quantification of DHE staining. The values represent means ± SEM. *P <* *.05, **P <* *.01, ***P <* *.001. Scare bar, 100 μm

Previous study showed that TGF‐β1 could up‐regulate the expression of collagen gene via ROS overproduction, this process eventually triggered HSCs activation and liver fibrogenesis.^33^ Firstly, we examined the relative mRNA expression level of ALDH2 in both rat primary hepatocytes and HSCs. The results showed that the mRNA expression level of ALDH2 in HSCs was relatively lower than that in hepatocytes (Figure 5A). Then, we used immunofluorescence staining to detect the co‐localization of ALDH2 and HSC markers (GFAP and α‐SMA) in the liver of WT mice after CCl_4_ challenge. The results revealed an activated state of HSCs and co‐localization of ALDH2 and GFAP/α‐SMA (Figure 5B,C), which indicated a specific role of ALDH2 in regulating HSCs function. To mimic CCl_4_‐induced liver fibrosis in vitro, HSC‐T6 cell line was used and treated with TGF‐β1. Besides, lentiviral vectors were employed to deliver ALDH2 or inhibit the expression of ALDH2 to further examine the effect of ALDH2 on hepatic fibrosis in vitro. Western blot data showed that the expression of ALDH2 was significantly knocked down or overexpressed by lentivirus transfection (Figure 6A). TGF‐β1 inducing assay was conducted at different concentrations (0, 1, 2, 5, 10 and 15 ng/mL) to activate HSCs. The expression of α‐SMA was used to represent the activation status of HSCs, and the results showed that the concentration of 15 ng/mL was most effective in activating HSCs, along with most significantly increased α‐SMA expression (Figure 6B). Then, we used 15 ng/mL TGF‐β1 to stimulate HSCs after transfected with ALDH2 knockdown (KD) or ALDH2 overexpression (OE) lentivirus vectors. The results showed that TGF‐β1 treatment significantly increased the expression of α‐SMA in NC and KD group, while showed no significant effect in OE group (Figure 6C,D). In addition, ALDH2 knockdown further increased the expression of α‐SMA after TGF‐β1 treatment, compared with NC group (Figure 6C,D).

Considering the pivotal role of ROS production in the pathogenesis of HSCs activation, we further examine the effect of Alda‐1, a potent ALDH2 activator, on attenuating TGF‐β1 treatment induced ROS production. DHE staining results showed increased ROS production after TGF‐β1 treatment (Figure 6E,F). ALDH2 knockdown further increased ROS generation, whereas ALDH2 overexpression showed no significant difference compared with the control group. Interestingly, Alda‐1 administration significantly reversed the overproduction of ROS induced by TGF‐β1 (Figure 6E,G). Furthermore, ALDH2 knockdown totally blocked the effect of Alda‐1 on attenuating ROS generation (Figure 6F,H), which indicated that the protective effect of Alda‐1 was dependent on ALDH2 activation. Taken together, our results indicated that Alda‐1 may reduce TGF‐β1‐induced ROS production and inhibit HSCs activation through ALDH2 activation.

### 3.6 Alda‐1 alleviates CCl_4_‐induced liver fibrosis and collagen deposition

**Figure 7:**
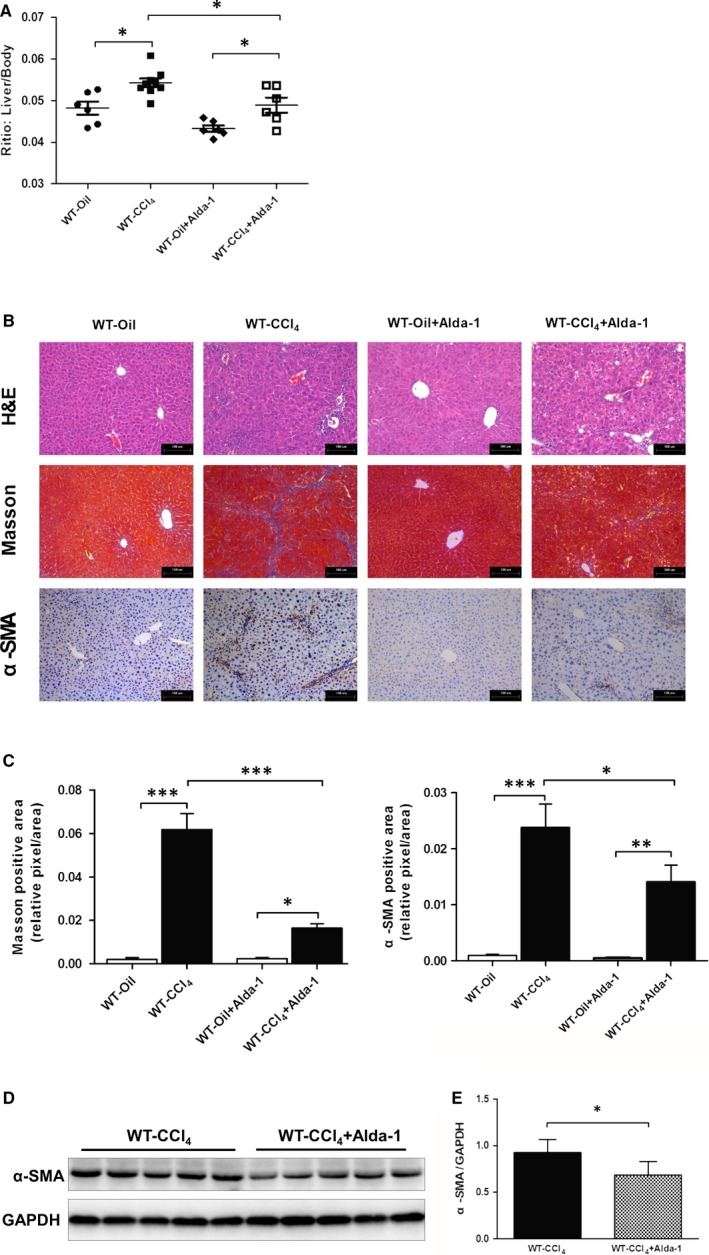
Alda‐1 alleviated chronic CCl_4_‐induced liver fibrosis. A, Liver/bodyweight ratio. B, Representative histopathological pictures of the liver. Upper panel: Representative H&E staining. Middle panel: Representative Masson's trichrome staining. Lower panel: Representative images of α‐SMA staining. C, Quantification of Masson's trichrome and α‐SMA‐positive area. D, Representative western blot pictures of α‐SMA. E, Quantification analyses of the expression of α‐SMA. The values represent means ± SEM (n = 6‐9). *P <* *.05, **P <* *.01, ***P <* *.001. Scare bar, 100 μm

To further determine the protective effect of ameliorating liver fibrosis, CCl_4_‐induced liver fibrosis model was conducted in WT mice. As shown in Figure 7A, Alda‐1 administration (depicted as WT‐CCl_4_+Alda‐1) significantly reduced the liver/bodyweight ratio compared with WT‐CCl_4_ mice. H&E staining showed that Alda‐1 administration reduced necrosis area and inflammatory infiltration compared with non‐Alda‐1‐treated WT‐CCl_4_ mice (Figure 7B). The Masson's trichrome and α‐SMA immunohistochemical staining showed that collagen deposition was significantly increased after CCl_4_ treatment, which was partly reversed after Alda‐1 administration. Additionally, western blot result showed that Alda‐1 administration significantly reduced α‐SMA expression compared with WT‐CCl_4_ group (Figure 7D,E).

### 3.7 Alda‐1 activates Nrf2/HO‐1 antioxidant pathway and Parkin‐related mitophagy

**Figure 8:**
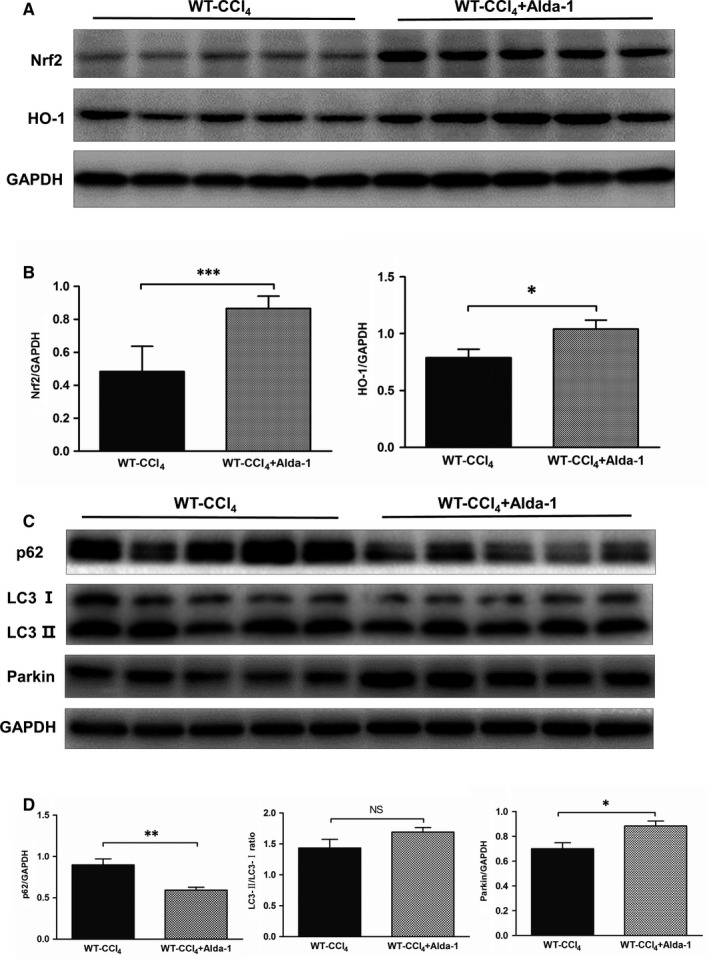
Alda‐1 activated the Nrf‐2/HO‐1 antioxidant pathway and Parkin‐mediated mitophagy. A, Representative western blot pictures of Nrf‐2 and HO‐1. B, Quantification analyses of the expression of Nrf‐2 and HO‐1. The values represent means ± SEM (n = 6‐9). *P <* *.05, **P <* *.01, ***P <* *.001. Scare bar, 100 μm. C, Representative western blot pictures of p62, LC3 and Parkin. D, Quantification analyses of the expression of p62, LC3 and Parkin. The values represent means ± SEM (n = 6‐9). *P <* *.05, **P <* *.01, ***P <* *.001. Scare bar, 100 μm

Previous results suggested that ALDH2 activation might ameliorate liver fibrosis via alleviating ROS generation. To determine whether Alda‐1 could act through activating the antioxidant system, we detected both Nrf2/HO‐1 antioxidant pathway and Parkin‐related mitophagy, which play a pivotal role in regulating oxidative stress. Western blot results revealed that Alda‐1 administration could significantly increase the protein expression levels of both Nrf2 and HO‐1 (Figure 8A,B), which indicated the activation of Nrf2/HO‐1 antioxidant pathway. Moreover, the protein expression level of p62 decreased, whereas Parkin increased after Alda‐1 treatment (Figure 8C,D), which indicated the activation of Parkin‐related mitophagy. Taken together, these data indicated that Alda‐1 may reduce liver fibrogenesis through activation of Nrf2/HO‐1 pathway and mitophagy in response to oxidative stress.

## 4 DISCUSSION

The salient findings of this study revealed that global ALDH2 deficiency aggravated liver injury and liver fibrosis induced by chronic CCl_4_ exposure, along with ROS overproduction, exacerbated hepatocyte apoptosis and inhibited mitophagy. In this study, we also focused on the potential protective role of Alda‐1, a selective activator of ALDH2, in liver fibrogenesis induced by CCl_4_ administration. Our results demonstrated that exogenously administered Alda‐1 can significantly attenuate liver fibrosis partly through up‐regulating the antioxidant Nrf2/HO‐1 pathway and activating Parkin‐related mitophagy. To our knowledge, this is the first report on the beneficial role of Alda‐1 in attenuating CCl_4_‐induced chronic liver fibrosis.

Liver fibrosis is a reversible early stage of liver cirrhosis,^4^ and currently no effective pharmacotherapy is available. It is well known that HSCs activation plays a vital role in the pathogenesis of liver fibrosis. Several factors including growth factors, cytokines, chemokines and oxidative stress could activate HSCs.^34^ Among these factors, endogenous ROS is a non‐negligible one, which is a key trigger to HSCs activation. Once activated, HSCs underwent transdifferentiation into α‐SMA‐positive myofibroblasts and produced substantial amounts of extracellular matrix proteins.^35^ ALDH2 is a key metabolic enzyme in the mitochondria, which not only metabolizes aldehyde into acetic acid, but also detoxifies toxic aldehyde substances such as 4‐HNE and MDA.^21^, ^22^ Several studies have revealed the protective role of ALDH2 in alcoholic liver cirrhosis and liver cancer.^36^, ^37^ Given that nearly half of the East Asian population possessed the loss‐of‐function mutation of ALDH2, the role of ALDH2 in liver fibrosis still needs further elucidation. In the present study, chronic CCl_4_ treatment induced higher protein expression of ALDH2. ALDH2 deficiency aggravated CCl_4_ treatment induced liver injury, showed as worse morphological change, increased ratio of liver weight to bodyweight, and increased levels of ALT and AST. Furthermore, ALDH2 deficiency induced more ROS production during detoxification of CCl_4_ and much higher expressions of α‐SMA and collagen 1α1 compared with CCl_4_‐induced WT mice, which suggested that ALDH2 deficiency exacerbates CCl_4_‐induced liver fibrogenesis. Whereas a study by Kwon et al showed a contradictory result that ALDH2 deficiency had no effect promoting liver fibrosis after CCl_4_ treatment compared to wild‐type mice.^38^ Given that a relatively low dose was used in their study, it would be reasonable to not have consistent results. As a main pro‐fibrotic cytokine, TGF‐β1 has been proved to induce the syntheses of α‐SMA and collagen 1α1 39 by HSC‐derived myofibroblasts through ROS accumulation. Our data showed that ALDH2 knockdown in vitro could further increase TGF‐β1‐induced ROS generation, and consequently promoted HSCs activation by increasing the expression of α‐SMA.

Previous work reported that increased ROS levels may influence Ca^2+^ homeostasis,^40^ which in turn caused an imbalance of Bcl‐2/Bax expression. The imbalance of anti‐apoptotic Bcl‐2 and pro‐apoptotic Bax determines the survival or death of hepatocyte following apoptosis stimulus.^41^ In our study, we found that CCl_4_ administration led to elevation of Bax while decrease in Bcl‐2 in hepatocytes. In other words, the significantly reduced ratio of Bcl‐2/Bax suggested that the chronic CCl_4_ exposure‐induced ROS overproduction could damage hepatocytes and liver tissue, thus leading to weakened anti‐apoptotic ability. Notably, ALDH2^−/−^ mice expressed much higher Bax and much lower Bcl‐2 than WT mice after CCl_4_ administration, which indicated that ALDH2 deficiency induced ROS overproduction could further promote hepatic cell apoptosis.

The protective role of ALDH2 prompted us to determine whether ALDH2 activation could ameliorate CCl_4_‐induced liver fibrosis in vivo. As a special activator of ALDH2, Alda‐1 was firstly discovered by Chen et al^42^ in 2008 and was proved to play a beneficial role in attenuating ischemia‐induced heart dysfunction. Our results revealed that Alda‐1 could significantly decrease TGF‐β1‐induced ROS production in vitro and alleviate CCl_4_‐induced liver fibrosis in vivo.

Mitochondrion is the main producer of ROS, which will in turn target mitochondria and cause mitochondrial damage.^43^ Studies have proved the critical role of mitochondrial homeostasis in maintaining hepatic function in the progression of liver diseases.^44^, ^45^ Autophagy is a metabolic process which involves in sensing oxidative stress and removing oxidatively damaged cytoplasmic organelles or cytosolic components.^46^ In particular, mitophagy appears to be critical in removing impaired mitochondria, thereby preventing ROS overproduction and cell damage.^47^ Mitophagy is activated by mitochondrial membrane depolarization, which induces the stabilization of PINK1 on the mitochondrial outer membrane and recruiting of Parkin from the cytosol. This initiates the localization of damaged mitochondria to membranes containing LC3, leading to the formation of autophagosomes, followed by fusion with lysosomes and clearance from cells.^48^ A previous study reported that mitophagy is impaired in the liver exposed to chronic CCl_4_, as evidenced by increased p62 expression and LC3‐II/LC3‐I ratio and decreased PINK1 and Parkin protein expression.^49^ Consistently, we observed increased expression of p62 and LC3‐II/LC3‐I ratio and decreased Parkin expression in the liver after chronic CCl_4_ treatment, which were further aggravated by ALDH2 deficiency, whereas ALDH2 activation by Alda‐1 significantly reversed the effect of CCl_4_.

Nrf2 is an important oxidative stress regulator which modulates HO‐1, GST, GCLC and GCLM.^50^, ^51^ Several studies have highlighted the beneficial role of antioxidants to protect against liver damage through activating the Nrf2/HO‐1 defence pathway.^52^, ^53^ In the present study, we found that CCl_4_ treatment could increase the Nrf2/HO‐1 cascade, which could be a compensatory effect of CCl_4_ treatment. Notably, ALDH2 deficiency partly reversed the up‐regulation of HO‐1 after CCl_4_ challenge, whereas administration of Alda‐1 could up‐regulate Nrf2/HO‐1 pathway as well as significantly decreased α‐SMA protein expression, which shed light on the application of Alda‐1 in clinical liver diseases.

## 5 CONCLUSION

In summary, our findings demonstrated that ALDH2 plays a vital role in CCl_4_‐induced mouse liver fibrosis model. Alda‐1, a pharmacological activator of ALDH2, alleviates CCl_4_‐induced liver fibrogenesis by activating Nrf2/HO‐1 antioxidant pathway and Parkin‐related mitophagy. These data provide a better understanding of ALDH2 in the pathogenesis of liver fibrosis and indicate that Alda‐1 may serve as a potential therapeutic molecule chemical.

## CONFLICTS OF INTEREST

The authors declare that there is no conflict of interest regarding the publication of this paper.

## AUTHORS' CONTRIBUTIONS

Xin Ma, Qin Luo and Hong Zhu conceived the study. Xin Ma, Qin Luo and Aijun Sun designed the study. Xin Ma, Qin Luo, Xuejing Liu and Kaili Zhang performed the study. Zhen Dong and Hong Zhu contributed to the figures and statistical analyses. Xin Ma drafted the manuscript. Qin Luo, Hong Zhu and Jian Wu revised the manuscript. Aijun Sun, Yunzeng Zou and Junbo Ge participated in the discussion and provided suggestions. All authors have read and approved the final manuscript.
